# Integrated analysis of the voltage-gated potassium channel-associated gene KCNH2 across cancers

**DOI:** 10.1186/s12859-023-05180-9

**Published:** 2023-02-15

**Authors:** Zequn Zheng, Yongfei Song

**Affiliations:** 1 Department of Cardiovascular Medicine, First Affiliated Hospital of Shantou University Medical College, Shantou University, Shantou, 515000 China; 2 Ningbo Institute of Innovation for Combined Medicine and Engineering, Lihuili Hospital Affiliated to Ningbo University, No. 378 Dongqing Road, Yinzhou District, Ningbo, 315000 Zhejiang China; 3 Department of Cardiovascular, Lihuili Hospital Facilitated to Ningbo University, Ningbo University, Ningbo, 315211 Zhejiang China

**Keywords:** KCNH2, Pancancer, Diagnosis, Prognosis, RNA modification, Immunoassay, Signalling pathways

## Abstract

KCNH2 encodes the human ether-a-go-go-related gene (hERG) potassium channel and is an important repolarization reserve for regulating cardiac electrical activity. Increasing evidence suggests that it is involved in the development of various tumours, yet a thorough analysis of the underlying process has not been performed. Here, we have comprehensively examined the role of KCNH2 in multiple cancers by assessing KCNH2 gene expression, diagnostic and prognostic value, genetic alterations, immune infiltration correlations, RNA modifications, mutations, clinical correlations, interacting proteins, and associated signalling pathways. KCNH2 is differentially expressed in over 30 cancers and has a high diagnostic value for 10 tumours. Survival analysis showed that high expression of KCNH2 was associated with a poor prognosis in glioblastoma multiforme (GBM) and hepatocellular carcinoma (LIHC). Mutations and RNA methylation modifications (especially m6A) of KCNH2 are associated with its expression in multiple tumours. KCNH2 expression is correlated with tumour mutation burden, microsatellite instability, neoantigen load, and mutant-allele tumour heterogeneity. In addition, KCNH2 expression is associated with the tumour immune microenvironment and its immunosuppressive phenotype. KEGG signalling pathway enrichment analysis revealed that KCNH2 and its interacting molecules are involved in a variety of pathways related to carcinogenesis and signal regulation, such as the PI3K/Akt and focal adhesion pathways. Overall, we found that KCNH2 and its interaction molecular are expected to be immune-related biomarkers for cancer diagnosis and prognosis evaluation, and are potential regulatory targets of singalling pathways for tumour development due to their significant role in cancers.

**Supplementary Information:**

The online version contains supplementary material available at 10.1186/s12859-023-05180-9.

## Introduction

According to the World Health Organization, cancer remains a leading cause of death worldwide. Multiple types of ion channels, such as potassium, calcium, and chloride channels, have been reported to be an essential part of tumour development [1]. They are often involved in many hallmarks of cancers, such as migration, invasion, proliferation, angiogenesis, and resistance to apoptosis [2]. A large number of literature reviews have been focused on this topic. The most notable channels are potassium channels, particularly the Eag1 channel and Erg1 (hERG) channel of the voltage-gated channel EAG family [3–6].

Encoded by the KCNH2 gene localized on 7q35-36, the hERG potassium channel generates an inwards delayed rectifier potassium current that is an important repolarizing current in phase 3 of the myocardial action potential and is the causal target of long QT syndrome (LQTS)-induced lethal arrhythmias [7]. While KCNH2 expression is generally thought to be restricted to the adult brain and heart, there is growing evidence that it is expressed in smooth muscle tissue as well as in cancer cells of various histologies [7]. This channel is overexpressed in 71% of tumours and cancer cell models from the neuroblast, glial, liver, lung, breast, ovary, cervix, prostate, gastrointestinal system, myeloid leukaemia, and retinoblastoma [8]. Three major functions associated with tumour cell biology can be attributed to hERG channel activity: (i) control of cell proliferation; (ii) regulation of tumour cell invasiveness, possibly through interaction with β1-integrin; and (iii) control of tumour cell neoangiogenesis by regulating angiogenic factor secretion [9]. More importantly, alterations in KCNH2 expression and function can occur at the genomic, transcriptional, posttranslational, and epigenetic levels [10–12]. Consequently, any of these modifications to hERG expression may regulate tumour development. As an example, the Sig1R protein regulates hERG channel expression through a posttranslational mechanism in leukaemic cells [12]. In addition, although specific mutations in the ion channel-encoding genes have not been identified, hERG mutations are associated with paediatric acute myeloid leukaemia [13]. More recently, high-throughput exome sequencing revealed that out of 2480 Japanese cancer patients, 36 patients had KCNH2 mutations [14].

hERG has gradually been proven to have diagnostic, prognostic, and therapeutic value as a target in a variety of tumours, particularly glioblastoma. However, a comprehensive analysis of KCNH2 across cancers is currently lacking. Here, we explored the differential expression, diagnostic and prognostic value, RNA modifications, mutations, and immune implications of KCNH2 across cancers. Finally, we also explored the role of KCNH2-associated genes in tumours.

## Methods and materials

### Expression analysis and diagnostic potential of KCNH2 across cancers

From the UCSC (https://xenabrowser.net/) database, we downloaded the standard pancancer dataset TCGA TARGET GTEx (PANCAN, N = 19,131, G = 60,499) and further extracted the expression data of the ENSG00000055118 (KCNH2) gene for each sample. Each expression value was log2(x + 1)-transformed, and finally, cancers with fewer than 3 samples were excluded, yielding a dataset involving 34 cancer types. Differential expression between normal and tumour samples in each cancer was analysed using R software. The diagnostic potential of KCNH2 in diverse cancers was estimated using receiver operating characteristic (ROC) analysis through the 'pROC' software package.

### The prognostic value of KCNH2 expression

A high-quality TCGA prognosis dataset from a previously published TCGA prognosis study in Cell [15], supplemented by TARGET follow-up data from UCSC and excluding samples with a follow-up time shorter than 30 days, was included for analysis. Expression data for 44, 38, 32, and 38 carcinomas and survival data for the corresponding samples were used for overall survival (OS), disease-specific survival (DSS), disease-free interval (DFI), and progression-free interval (PFI) analyses.

The coxph function in the R package survival (version 3.2) was adapted to build a Cox proportional hazards regression model to analyse the prognostic relationship between KCNH2 expression and each tumour. The optimal cut-off value for KCNH2 was calculated by the R package maxstat, and the patients were divided into two high and low groups. The log-rank test was used to determine prognostic significance.

### Analysis of RNA modification-related genes

The expression data of the KCNH2 gene and 44 genes related to three types of RNA modifications (m1A(10), m5C(13), and m6A(21)) in different tumour samples were extracted, and their Pearson correlations were then calculated.

### KCNH2 mutation and expression analysis

The single nucleotide variation (SNV) and copy number variation (CNV) datasets (level 4 datasets) of all TCGA samples processed by MuTect2 [16] and GISTIC [17] software, respectively, were downloaded from the Genomic Data Commons (GDC) (https://portal.gdc.cancer.gov/). SNVs for 17 cancer species and CNVs for 32 cancer species were obtained. The differential expression of genes in each tumour in variant and nonvariant samples was further analysed. Structural domain information of the KCNH2 protein was obtained from the R package maftools, and SNV data were integrated to map the mutation landscape.

### Analysis of genomic heterogeneity and KCNH2 expression

The SNV information of KCNH2 was used to calculate the tumour mutation burden (TMB) and mutant-allele tumour heterogeneity (MATH) for each tumour using the tmb function and inferHeterogeneity function of the R package maftools, respectively. In addition, we obtained microsatellite instability (MSI) scores [18] and neoantigen load (NAL) data [19] from previous studies. We assessed the Spearman correlation between KCNH2 expression and these parameters in each tumour type.

### Analysis of immune infiltration scores, immune cells, and immune checkpoints

Based on KCNH2 expression (log2(x + 0.001) transformed), the R package ESTIMATE (version 1.0.13) was used to calculate the stromal, immune, and ESTIMATE scores for each tumour. The "Timer" method of the R package IOBR was used to assess the infiltration scores of B cells, CD4^+^ T cells, CD8^+^ T cells, macrophages, neutrophils, and dendritic cells; the EPIC method in the IOBR package was used to further assess the infiltration scores of B cells, cancer-associated fibroblasts (CAFs), CD4^+^ T cells, CD8^+^ T cells, endothelial cells, macrophages, NK cells, and other cells. The Spearman's correlation coefficient between gene expression and immune infiltration score was calculated.

For immune checkpoint analysis, the Pearson correlations between KCNH2 expression and the expression of 60 marker genes of both types of immune checkpoint pathways (inhibitory (24) and stimulatory (36)) were assessed.

### Clinical staging and gene expression

Differences in gene expression between patients with each tumour grouped by clinical stage (T, N, M, stage I-IV) and sex were assessed by unpaired Wilcoxon rank sum and signed rank tests. Spearman's correlation coefficient between KCNH2 expression and age was further calculated using the corr.test function in R package psych (version 2.1.6).

### KEGG signalling pathway analysis of KCNH2 interacting proteins

The KCNH2 interacting protein network information from the STRING and GeneMANIA databases was extracted, and the network was visualized using Cytoscape software (version 3.9). The involved molecules were subjected to enrichment analysis with the R package clusterProfiler to obtain gene set KEGG enrichment results [20, 21]. Gene set size was set to a minimum of 5 and a maximum of 5000, and a *P* value of < 0.05 and an FDR of < 0.25 were considered to indicate statistical significance.

### Statistical analysis

The significance of differences between the two pairs was analysed using the unpaired Wilcoxon rank sum and signed rank tests, and the Kruskal‒Wallis test was used to test for differences in multiple samples. Unless otherwise stated, *P* < 0.05 was considered to indicate statistical significance.

## Results

### KCNH2 is aberrantly expressed in a variety of tumours with favourable diagnostic value

**Fig. 1:**
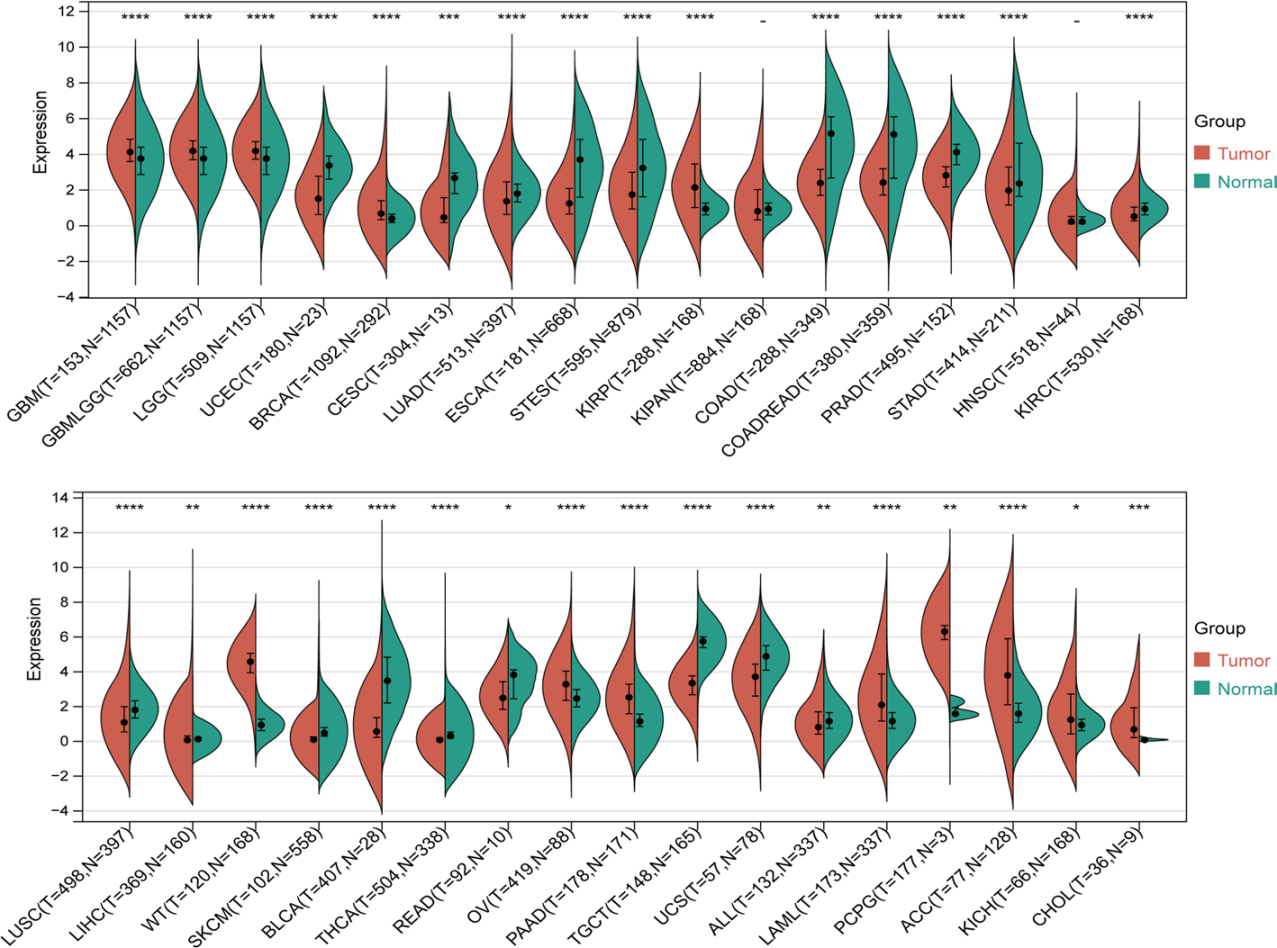
Differential expression of KCNH2 in tumour versus normal samples for 34 tumour types. GBM, glioblastoma multiforme; GBMLGG, glioma; LGG, brain lower grade glioma; UCEC, uterine corpus endometrial carcinoma; BRCA, breast invasive carcinoma; CESC, cervical squamous cell carcinoma and endocervical adenocarcinoma; LUAD, lung adenocarcinoma; ESCA, oesophageal carcinoma; STES, stomach and oesophageal carcinoma; KIRP, kidney renal papillary cell carcinoma; KIPAN, pan-kidney cohort (KICH + KIRC + KIRP); COAD, colon adenocarcinoma; COADREAD, colon adenocarcinoma/rectum adenocarcinoma oesophageal carcinoma; PRAD, prostate adenocarcinoma; STAD, stomach adenocarcinoma; HNSC, head and neck squamous cell carcinoma; KIRC, kidney renal clear cell carcinoma; LUSC, lung squamous cell carcinoma; LIHC, liver hepatocellular carcinoma; WT, high-risk Wilms tumour; SKCM, skin cutaneous melanoma; BLCA, bladder urothelial carcinoma; THCA, thyroid carcinoma; READ, rectum adenocarcinoma; OV, ovarian serous cystadenocarcinoma; PAAD, pancreatic adenocarcinoma; TGCT, testicular germ cell tumours; UCS, uterine carcinosarcoma; ALL, acute lymphoblastic leukaemia; LAML, acute myeloid leukaemia; PCPG, pheochromocytoma and paraganglioma; ACC, adrenocortical carcinoma; KICH, kidney chromophobe; CHOL, cholangiocarcinoma. *P < 0.05, **P < 0.01, ****P < 0.0001, ^−^ no significance

**Fig. 2:**
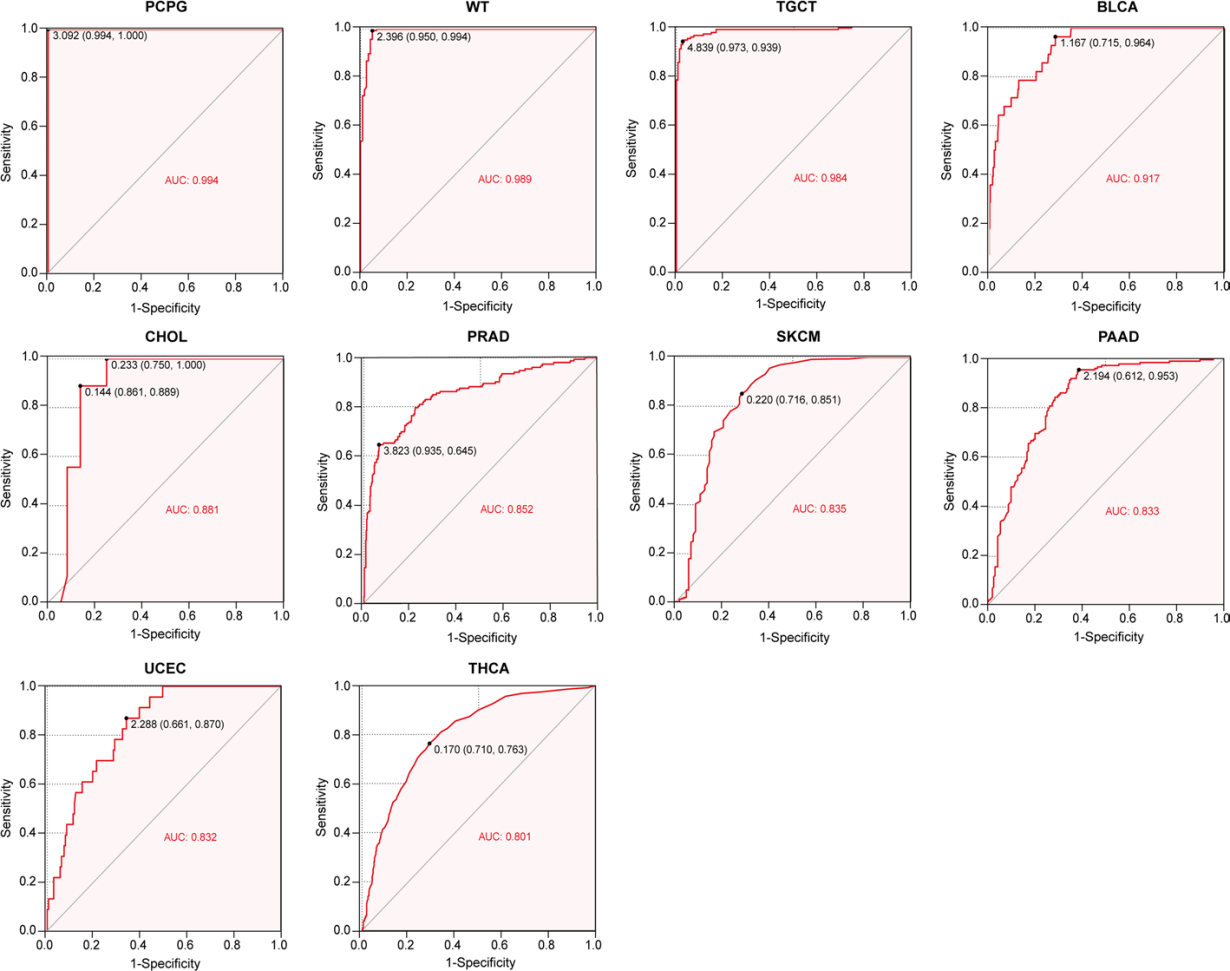
The diagnostic value of KCNH2 in diverse cancers evaluated by the receiver operating characteristic (ROC) curve. An area under the receiver operating characteristic curve (AUC) value over 0.8 is considered to indicate a good predictive effect. The coordinates on the curves represent the best diagnostic cut-off values for KCNH2 expression and the respective 95% confidence intervals

Through differential expression analysis of normal and tumour samples for 34 cancers, we observed significant upregulation of KCNH2 in 14 tumour datasets, including those for glioblastoma multiforme (GBM), glioblastoma and low-grade glioma (GBMLGG), brain lower grade glioma (LGG), breast invasive carcinoma (BRCA), kidney renal papillary cell carcinoma (KIRP), liver hepatocellular carcinoma (LIHC), Wilms tumour (WT), ovarian serous cystadenocarcinoma (OV), pancreatic adenocarcinoma (PAAD), acute myeloid leukaemia (LAML), pheochromocytoma and paraganglioma (PCPG), adrenocortical carcinoma (ACC), kidney chromophobe (KICH), and cholangiocarcinoma (CHOL) (Fig. 1). To further validate the value of differential gene expression in the diagnosis of tumours, the area under the ROC curve (AUC) value was assessed. Diagnostic performance according to the AUC values was good to excellent for 10 tumours [in order of the diagnostic value of KCNH2: PCPG, WT, testicular germ cell tumour (TGCT), bladder urothelial carcinoma (BLCA), CHOL, prostate adenocarcinoma (PRAD), skin cutaneous melanoma (SKCM), PAAD, uterine corpus endometrial carcinoma (UCEC), and thyroid carcinoma (THCA)] (Fig. 2).

### Identification of the prognostic value of KCNH2 in pancytopenia

**Fig. 3:**
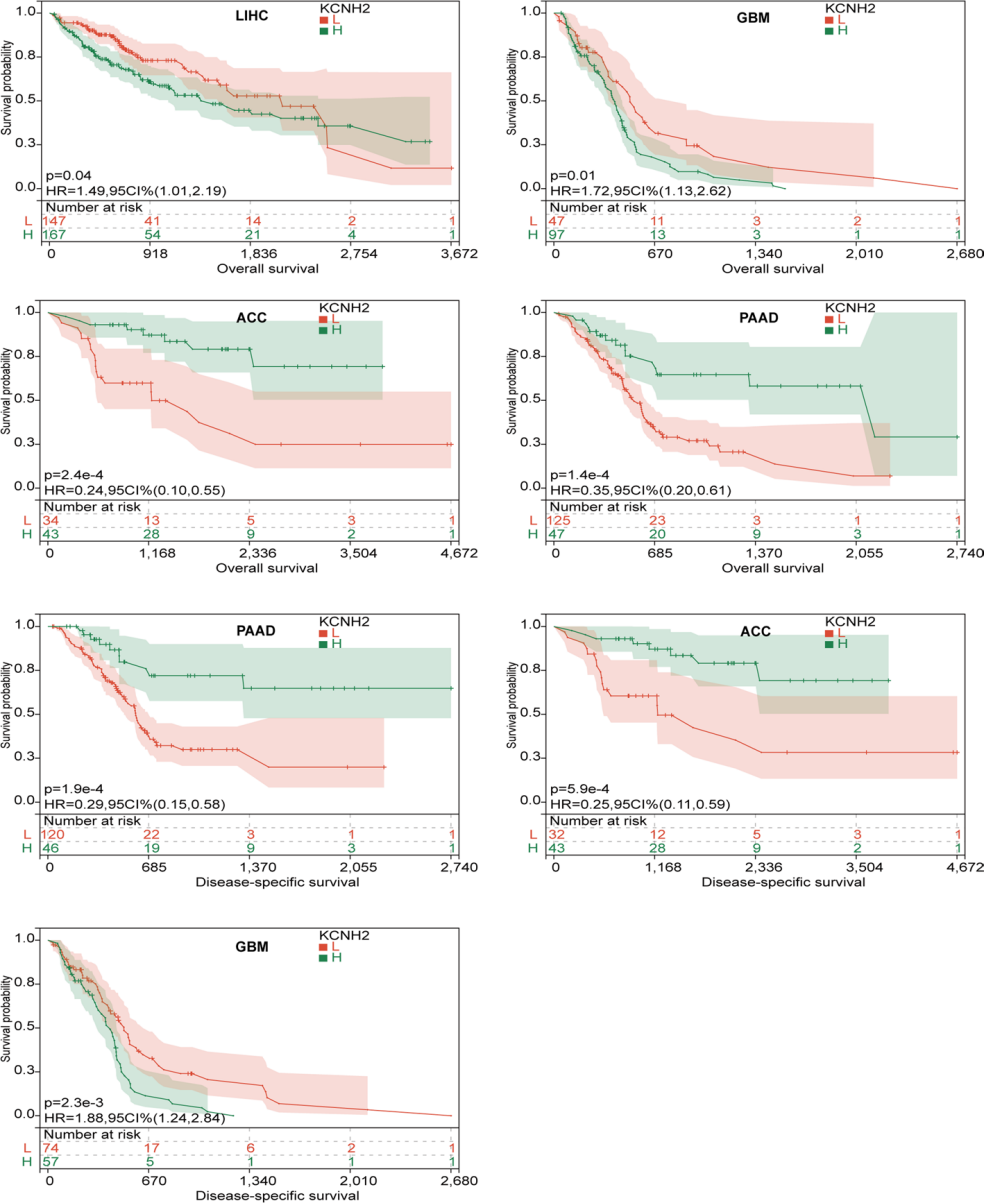
Overall survival (OS) and disease-specific survival (DSS) of patients grouped based on KCNH2 expression in different cancers. Statistically significant carcinomas in the Cox regression analysis of OS and DSS were used for post hoc Kaplan‒Meier (KM) survival. HR, hazard ratio; 95% CI, 95% confidence interval

We further investigated the prognostic impact of KCNH2 in cancer patients by Cox risk regression proportional models. Forest plots generated by Cox regression of OS, DSS, DFI, and PFI indicated the survival impact of KCNH2 on patients (Additional file 1: Fig. S1). The results of OS and DSS analysis showed that high KCNH2 gene expression was associated with a poor prognosis in three tumour types [GBM (HR 1.20 in OS, HR 1.27 in DSS), LIHC (HR 1.34 in OS, HR 1.26 in DSS), and THCA (HR 1.90 in OS, HR 2.41 in DSS)], while low expression was associated with a poor prognosis in 2 tumour types [PAAD (HR 0.72 in OS, HR 0.70 in DSS) and ACC (HR 0.76 in OS, HR 0.78 in DSS)] (Additional file 1: Fig. S1). Post hoc Kaplan‒Meier (KM) survival curve analysis validated these results (Fig. 3).

### KCNH2 and RNA methylation modification genes across cancers

**Fig. 4:**
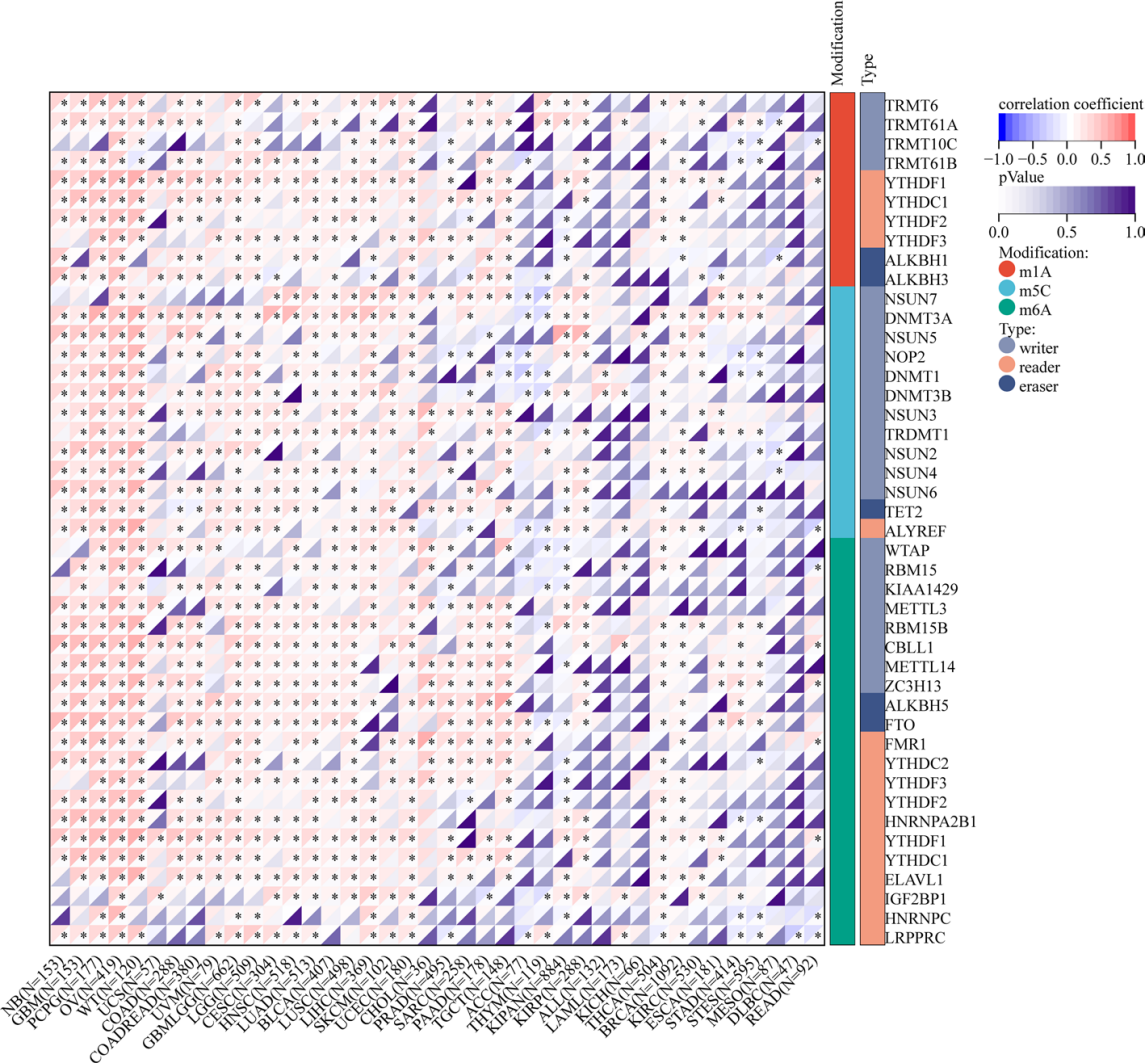
Correlation of KCNH2 expression with the levels of RNA modifications in m1A, m5C, and m6A across cancers. A positive correlation is denoted by red, whereas a negative correlation is indicated by blue. NB, neuroblastoma; UVM, uveal melanoma; SARC, sarcoma; THYM, thymoma; MESO, mesothelioma; DLBC, lymphoid neoplasm diffuse large B-cell lymphoma

RNA methylation modification pathways, particularly those related to N6-methyladenosine (m6A), are misregulated in human cancers, and alterations in the expression levels of writers, erasers, and readers of modifiers may significantly contribute to the altered gene expression observed in cancer, and thus, these regulators may be ideal targets for the development of cancer therapies. Here, we characterized the correlation of KCNH2 expression with the levels of 44 RNA modifications in m1A, m5C, and m6A (Fig. 4). We found that KCNH2 was most highly correlated with the 3 types of RNA modification in neuroblastoma (NB), GBM, PCPG, and OV. In addition, there was a significant correlation between KCNH2 expression and m6A level in various tumours, such as colon adenocarcinoma (COAD), uveal melanoma (UVM), and LGG. m6A is removed by demethylases such as FTO and ALKBH5 [22], whose expression levels were significantly correlated with those of KCNH2 in PRAD, sarcoma (SARC), PAAD, and TGCT (Fig. 4).

### Differential KCNH2 mutation and expression across cancers

**Fig. 5:**
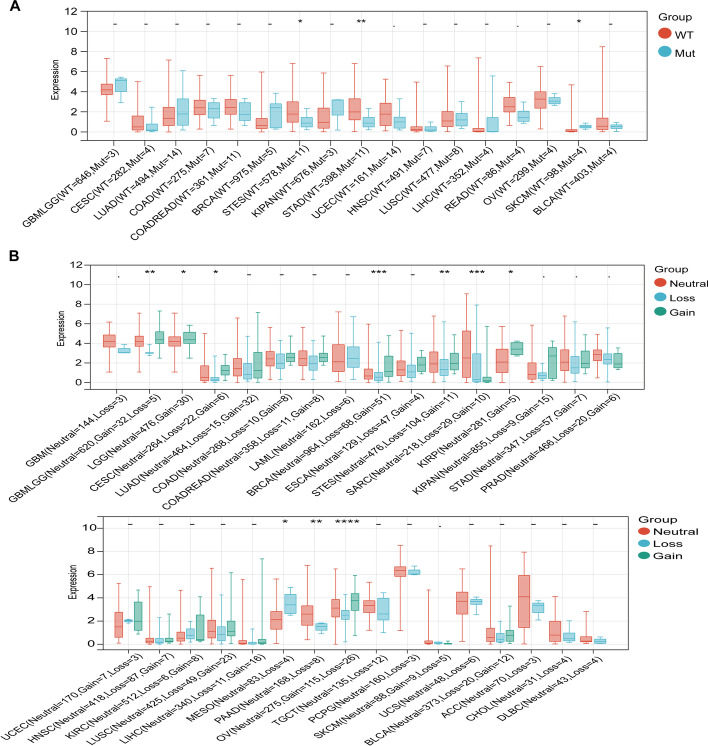
The variant profile of KCNH2 across cancers. (**A**) Single nucleotide variation (SNV) in KCNH2 for 17 cancer species. WT, nonvariant samples; Mut, variant samples. (**B**) Copy number variation (CNV) of KCNH2 in 32 cancers. *P < 0.05, **P < 0.01, ***P < 0.001, ****P < 0.0001, – no significance

KCNH2 gene mutation may be related to the occurrence of tumours. We used the SNV data for KCNH2 and its structure to map the mutational landscape of tumours, and we found that a large number of missense mutations were concentrated in the transmembrane segments of the full-length structure of KCNH2 (Additional file 1: Fig. S2). We further calculated the differences in gene expression in each tumour in samples with and without SNV and observed significant differences in three tumours, including stomach and oesophageal carcinoma (STES), stomach adenocarcinoma (STAD), and SKCM (Fig. 5A). Furthermore, we obtained CNV data (classified as no change, CNV loss, and CNV gain) for tumour samples. KCNH2 was significantly differentially expressed in samples grouped by CNV type in 10 tumours (GBMLGG, LGG, cervical squamous cell carcinoma and endocervical adenocarcinoma (CESC), BRCA, STES, SARC, KIRP, mesothelioma (MESO), PAAD, OV) (Fig. 5B).

### Correlation of KCNH2 expression with tumour genomic heterogeneity

**Fig. 6:**
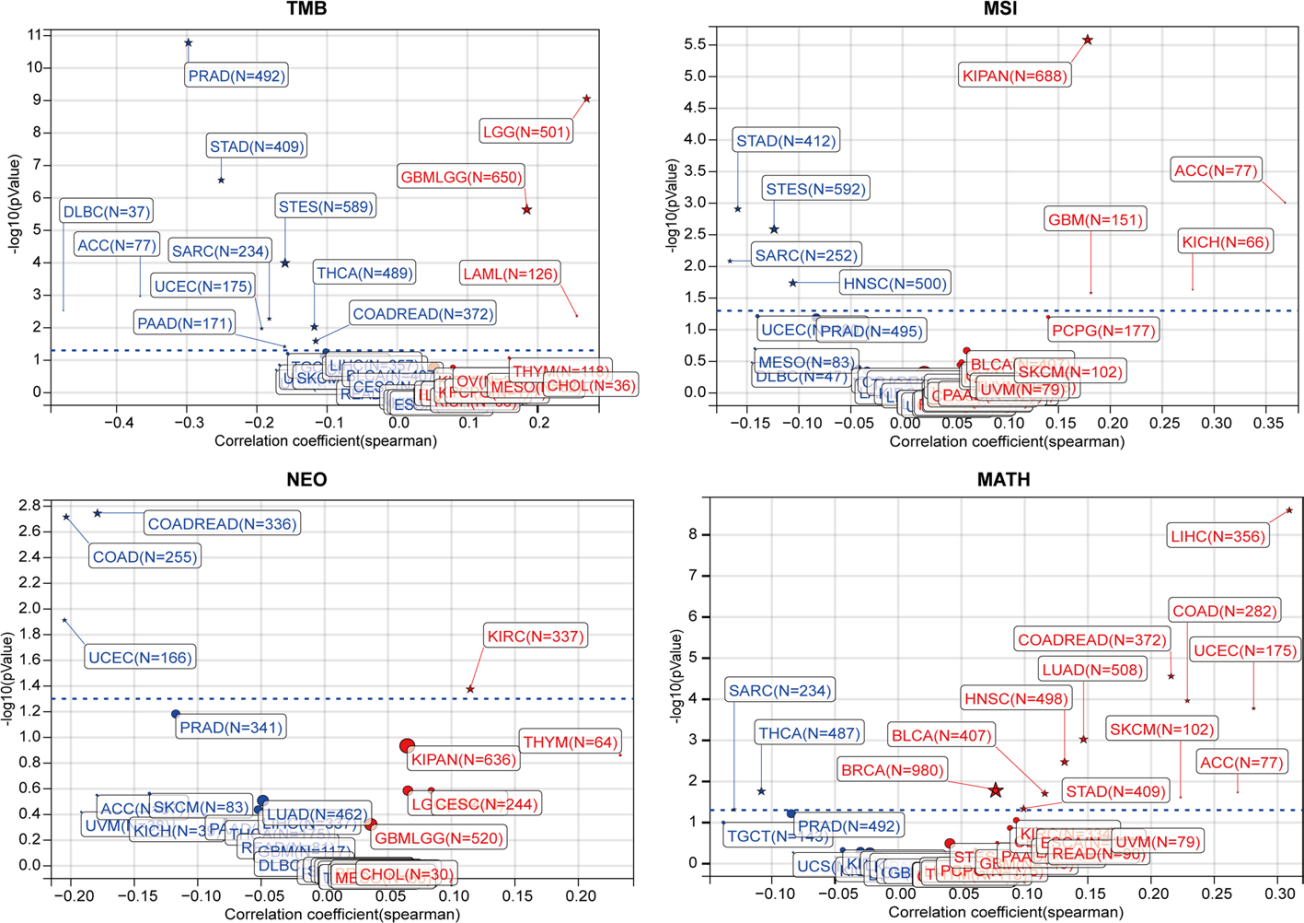
Correlation of KCNH2 expression with tumour genomic heterogeneity. Tumours with statistically significant (*P* < 0.05) correlations between KCNH2 expression and tumour features, with red indicating a positive association and blue a negative association. TMB, tumour mutation burden; MSI, microsatellite instability; NAL, neoantigen load; MATH, mutant-allele tumour heterogeneity

The heterogeneity of the tumour genome contributes to its tumour malignancy, marker expression, immunotherapy response, prognosis, etc., and has a substantial impact on its occurrence and development. Therefore, we analysed the correlation between TMB, MSI, MATH, NAL, and KCNH2 expression (Additional file 1: Table S1). In the analysis of MATH, representing malignancy, high KCNH2 expression was associated with increased MATH in 11 tumours (Fig. 6). However, high expression correlated with low TMB in 10 tumours. High TMB means that more tumour neoantigens (indicated by the NAL metric) can be generated and recognized by T cells, which may induce better immunotherapy effects in the tumour. NAL, a new metric for assessing the immunotherapy response, showed a negative correlation with high KCNH2 expression in COAD, COAD/rectum adenocarcinoma oesophageal carcinoma (COADREAD), and UCEC (Fig. 6).

### Tumour immune-features related to KCNH2

**Fig. 7:**
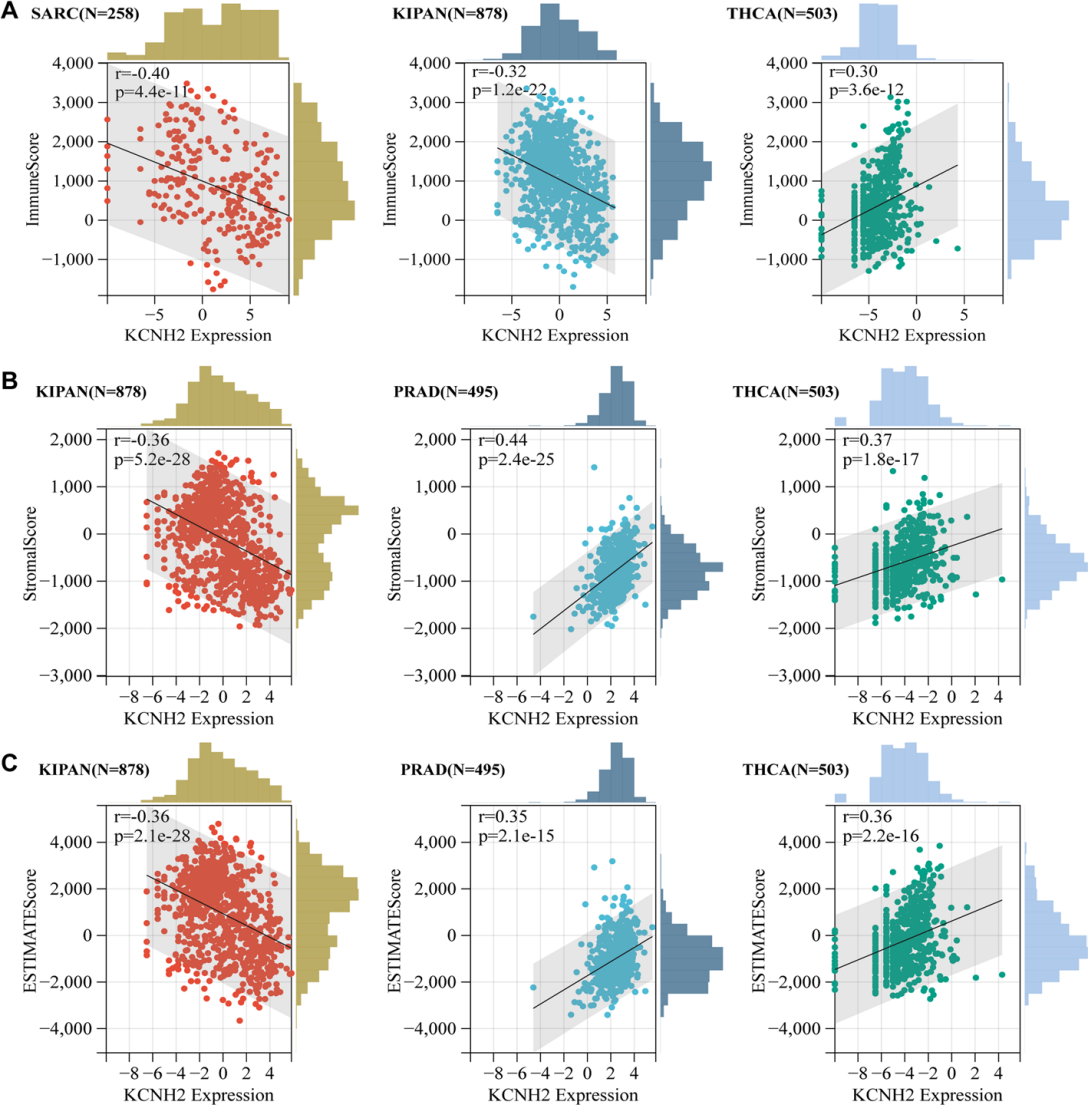
Association between KCNH2 expression and tumour immune infiltration score in the top three tumour types. (**A**) Immune score. (**B**) Stromal score. (**C**) ESTIMATE scores

**Fig. 8:**
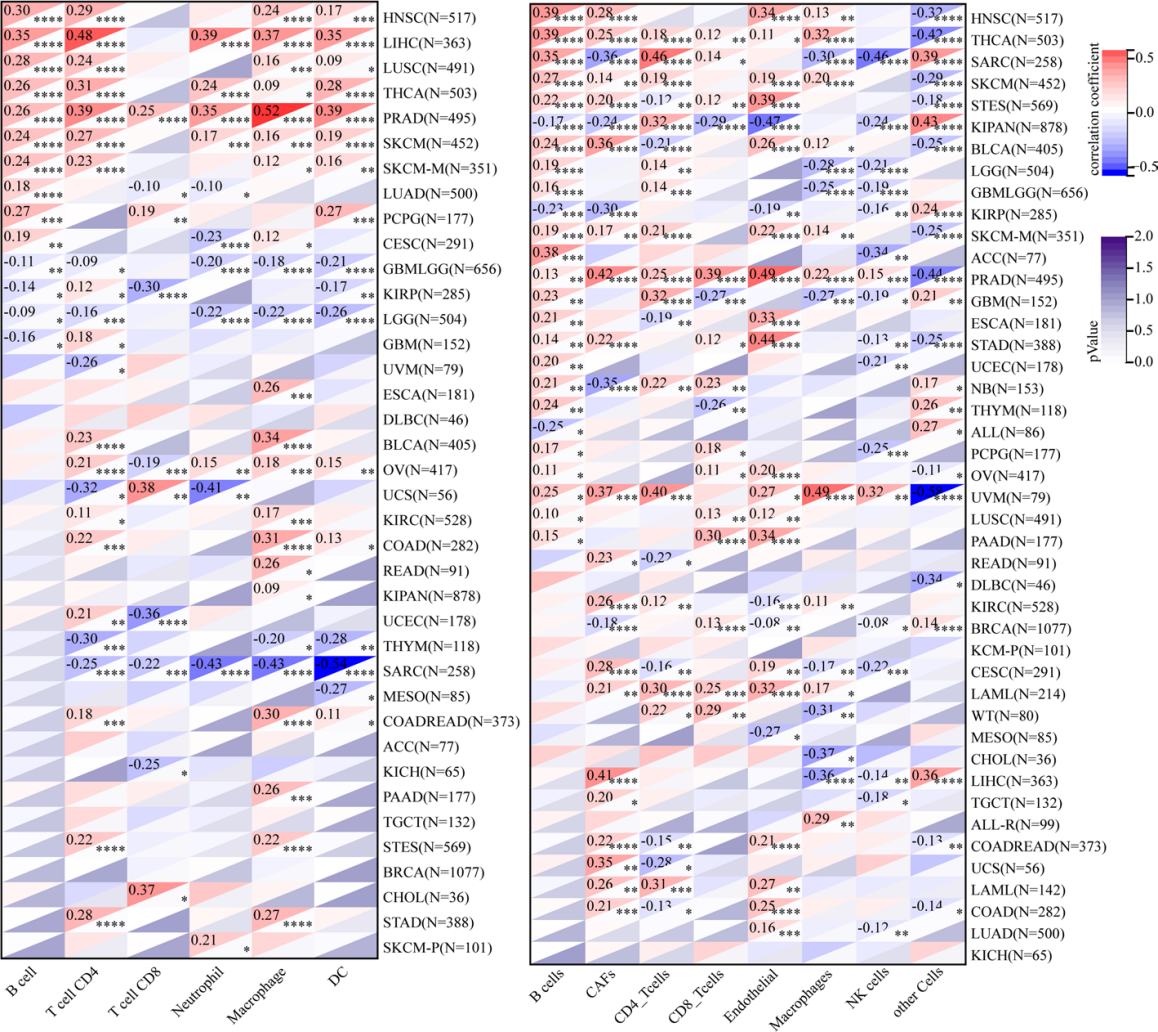
The level of immune cell infiltration across cancers. On the left, the "Timer" method is employed, and on the right, the "EPIC" method is employed. Red indicates a positive correlation, and blue indicates a negative correlation. *P < 0.05, **P < 0.01, ****P < 0.0001

**Fig. 9:**
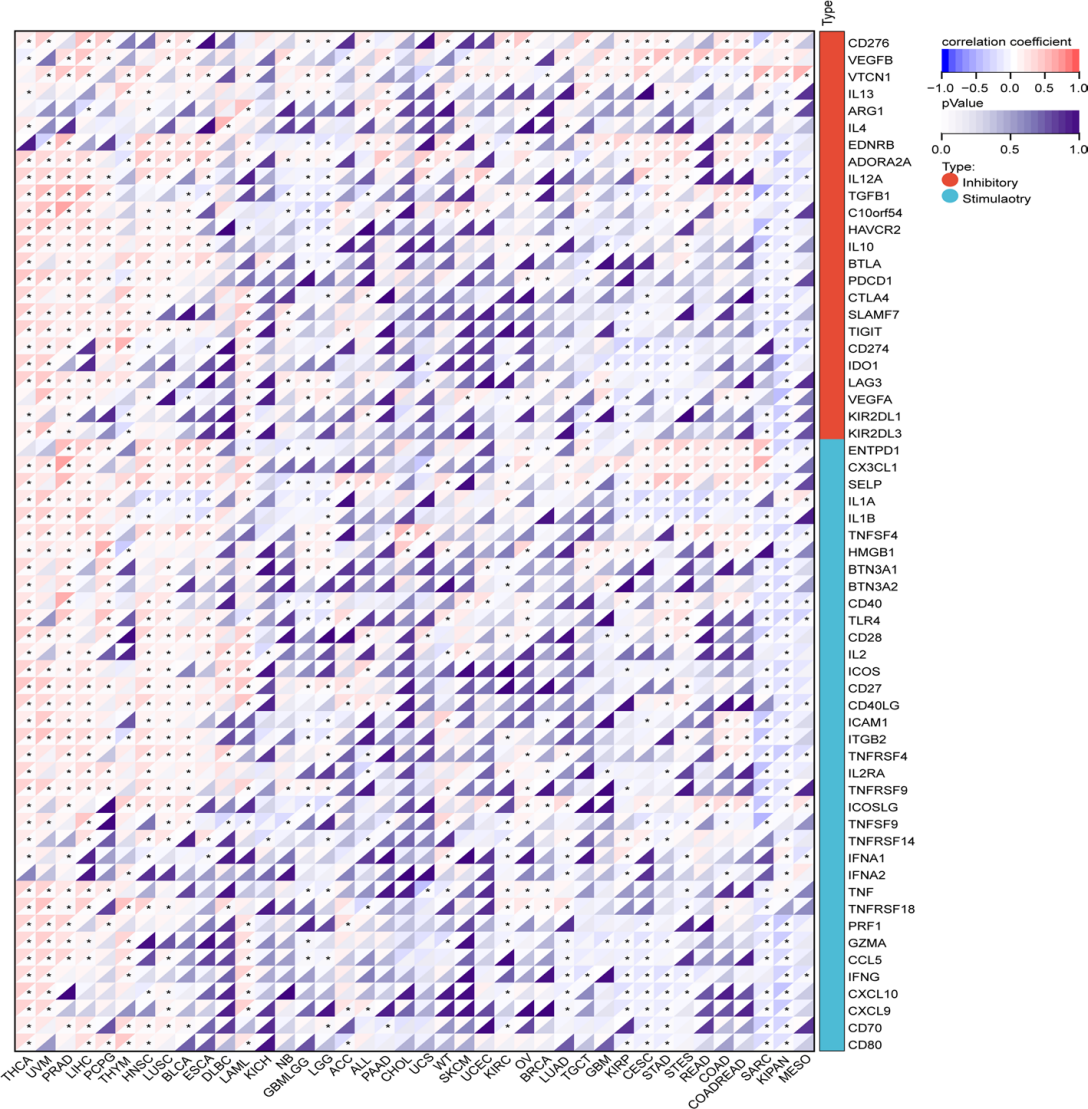
Correlation of KCNH2 expression with immune checkpoints. Red indicates a positive correlation, and blue indicates a negative correlation

Tumour-infiltrating immune cells greatly influence the immune microenvironment and affect the prognosis of the tumour. We first identified the relationship between KCNH2 expression and immune infiltration based on stromal, immune, and ESTIMATE scores (Additional file 1: Table S2). The tumours most significantly associated with KCNH2 expression were SARC, KIPAN, THCA, and PRAD (Fig. 7), and we further analysed the level of immune cell infiltration by the "Timer" method and the "EPIC" method. We observed a correlation between KCNH2 expression and immune cell infiltration, especially in B cells, in most of the tumour types (Fig. 8). We also evaluated the potential correlation of KCNH2 expression with 60 immune checkpoints and showed that KCNH2 was positively correlated with most immune checkpoints across cancers, especially THCA, UVM, and PRAD (Fig. 9).

### Differences in KCNH2 expression at different tumour stages

**Fig. 10:**
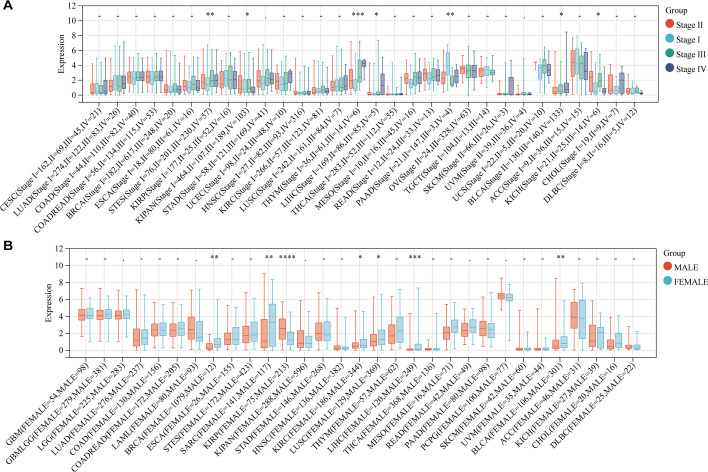
Expression of KCNH2 in cancer patients grouped by clinical stage and other characteristics. (**A**) Differences in KCNH2 expression in patients grouped by clinical stage I-IV. (**B**) Differences in KCNH2 expression in patients grouped by sex. *P < 0.05, **P < 0.01, ***P < 0.001, ****P < 0.0001, – no significance

We used clinical information from the samples to further characterize the differences in KCNH2 expression across tumour patient stages. First, when patients were grouped based on TMN stage, only BLCA had a significant difference in KCNH2 expression between groups (Additional file 1: Fig. S3). Then, we observed significant differences in gene expression in seven tumours, including STES, KIPAN, thymoma (THYM), LIHC, PAAD, BLCA, and KICH, grouped by clinical stage (Fig. 10A). Furthermore, we assessed the impact of sex, and seven tumours [BRCA, SARC, KIRP, kidney renal clear cell carcinoma (KIRC), lung squamous cell carcinoma (LUSC), LIHC, and BLCA] were detected to have differential expression of KCNH2 between males and females (Fig. 10B). Finally, we investigated the correlation between age and gene expression in the pancancer dataset, and we observed a significant positive correlation in three tumours: GBMLGG (R = 0.167, *P* < 0.001), LGG (R = 0.236, *P* < 0.001), and UCEC (R = 0.221, *P* < 0.005) (Additional file 1: Fig. S4).

### KEGG signalling pathway analysis of KCNH2-interacting proteins

**Fig. 11:**
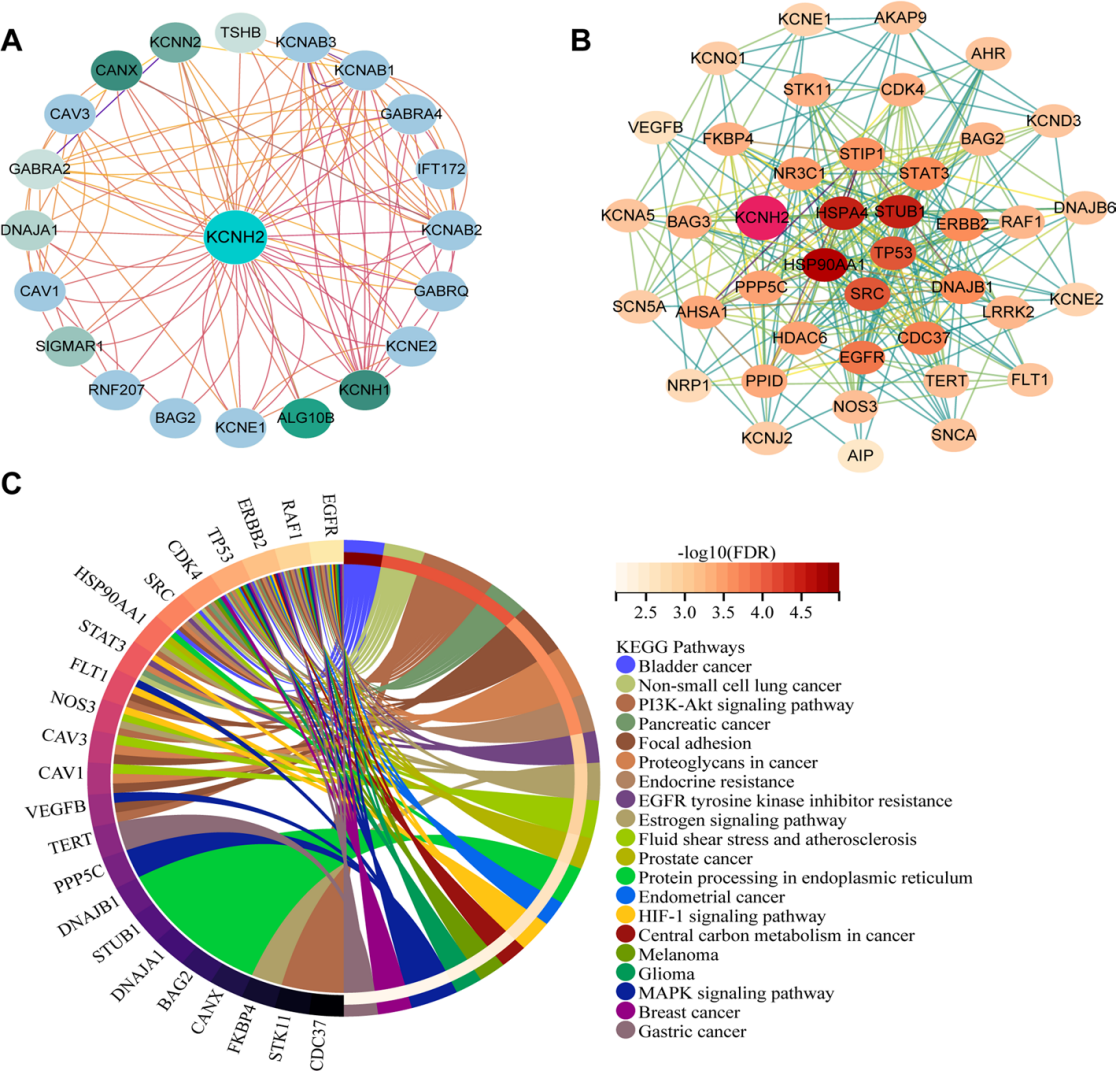
KEGG signalling pathway enrichment analysis of KCNH2-interacting proteins. (**A**) Twenty KCNH2-interacting factors derived from the GeneMANIA website. (**B**) The KCNH2 protein interaction network in STRING. The degree value of nodes is indicated using the colour scale. (**C**) KEGG signalling pathway enrichment analysis of combined molecules from **A** and **B**

To further understand the role of KCNH2 in regulating tumours, we performed KEGG signalling pathway enrichment analysis of KCNH2-interacting proteins derived from the GeneMANIA (Fig. 11A) and STRING websites (Fig. 11B). The results showed that these molecules were associated with multiple individual tumours, and 11 of the related genes, including RAF1, EGFR, TP53, ERBB2, HSP90AA1, NOS3, CDK4, VEGFB, FLT1, STK11, and CDC37, were enriched in the PI3K-Akt signalling pathway [20, 21]. In addition, several pathways closely related to tumour development, such as the FAK-related focal adhesion and MAPK pathways, were enriched un genes related to KCNH2 (Fig. 11C) [21].

## Discussion

The hERG potassium channel is widely recognized for its important role in the repolarization of myocardial action potential and as a target for a large number of cardiotoxic drugs. The ground-breaking research of DeCoursey, published in 1984 [23], showing the role of potassium channels in the proliferation of T lymphocytes, opened up new avenues for the study of ion channel biology. Our work was inspired by the finding that hERG is associated with pathological processes in a variety of tumours and the lack of analysis of its role across cancers. We found that KCNH2 was significantly differentially expressed in all 32 tumours and had good diagnostic value in 10 cancers. Consistent with the results of this study, the correlation of hERG expression with prognosis has also been shown in cell models of PRAD [24] and SKCM [25]. Furthermore, the KCNH2 gene influenced UCEC cell proliferation and regulated the migration of human anaplastic thyroid cancer cells [26, 27], a result consistent with our finding that THCA patients with high hERG expression had a poor prognosis. These results suggest that beyond its role in myocardial electrical activity, KCNH2 has great potential to become a tumour marker.

By analysing the relationship between KCNH2 expression levels and the levels of 3 types of RNA methylation modifications, we found that KCNH2 was associated with almost the expression levels of almost all assessed writers, erasers, and readers in the 4 tumour types NB, GBM, PCPG, and OV. Methylation modifications can control all aspects of the maturation of mRNAs, both in the nucleus and in the cytoplasm [28]. Among these modifiers, the most widely studied is m6A, which is particularly abundant in the brain and is essential for neurodevelopment. Recent studies have shown that the m6A binding protein RBM45 is required for NB cell differentiation [29], and in cancer stem cells generated from glioblastoma patients, PDGFR (platelet-derived growth factor receptor) activity exhibits enhanced m6A methylation, which encourages cell maintenance through control of mitophagy [30]. This evidence further reinforces the importance of posttranscriptional RNA modifications in cancer progression, but it remains unclear whether there is an impact on tumours through the methylation of KCNH2.

As previously mentioned, mutations in the KCNH2 gene may be associated with tumorigenesis. We observed differences in the expression of the mutation in gastrointestinal tumours STES and STAD. The expression of hERG has been described to decrease the resting membrane potential of cancer cells and restore polarization at the end of the G1 phase, thereby promoting cell cycle progression and leading to proliferation [31]. The altered activity of mutant hERG appears to explain the increased malignancy of the tumours, and in STAD, reduced levels of the regulatory β-subunit KCNE2 lead to an increased hERG current [32].

Differences in individual arrhythmia susceptibility are a pressing issue in exploring KCNH2 mutation-induced type 2 LQTS [33]. Here, we found that KCNH2 expression is generally higher in female tumour patients. Studies have suggested that oestradiol can increase hERG channel membrane trafficking and repolarization currents through enhanced oestradiol α receptor-mediated HSP90 interactions [34]. More importantly, numerous factors, such as temperature, hypoxia, pH, and potassium concentration, can influence hERG protein maturation [35]. Further clarification of the relationship between tumours, oestrogen, and hERG expression may provide new insights into the roles of KCNH2.

We also subjected the KCNH2-interacting proteins to KEGG pathway enrichment analysis. Surprisingly, we not only observed their enrichment in a variety of solid carcinomas, such as bladder cancer, non-small cell lung cancer, and PAAD but also revealed their enrichment in important tumour signalling axes, such as the PI3K-Akt and focal adhesion signalling pathways. The PI3K/Akt/mTOR signalling pathway affects several cellular processes, such as growth, proliferation, and survival [36]. Key factors in the pathway, particularly Akt, have emerged as potential therapeutic targets in diverse tumours. Moreover, the KCNH2 channel has been identified to be associated with the Wnt/β-catenin pathway in focal adhesion by interacting with β1-integrin to influence tumour invasiveness [37, 38]. The roles of 11 identified KCNH2-interacting proteins in tumours still need to be explored; however, targeted inhibition of heat shock proteins, such as HSP90, is not a new topic. In conclusion, this result not only provides new potential targets for intervention but also advances the understanding of the relevance of KCNH2 to tumours.

## Supplementary Information

**Additional file 1**. Supplemental data.

## Data Availability

All data supporting the findings of this study are included in this article (and its Additional file 1). The datasets analysed during the current study are available in the following online repositories: 1. https://xenabrowser.net/. 2. https://portal.gdc.cancer.gov/. 3. https://cn.string-db.org/. 4. http://genemania.org/
