# Supplementary material for: Integrated analysis of the voltage-gated potassium channel-associated gene KCNH2 across cancers
Source: BMC Bioinformatics. 2023 Feb 15;24:51. doi: 10.1186/s12859-023-05180-9 (PMC9933257; doi:10.1186/s12859-023-05180-9)
Supplement: Supplementary file 1 — Additional file 1. Supplemental data. [file 12859_2023_5180_MOESM1_ESM.docx]

## Integrated analysis of the voltage-gated potassium channel-associated gene KCNH2 across cancers

**SUPPLEMENTAL MATERIAL**


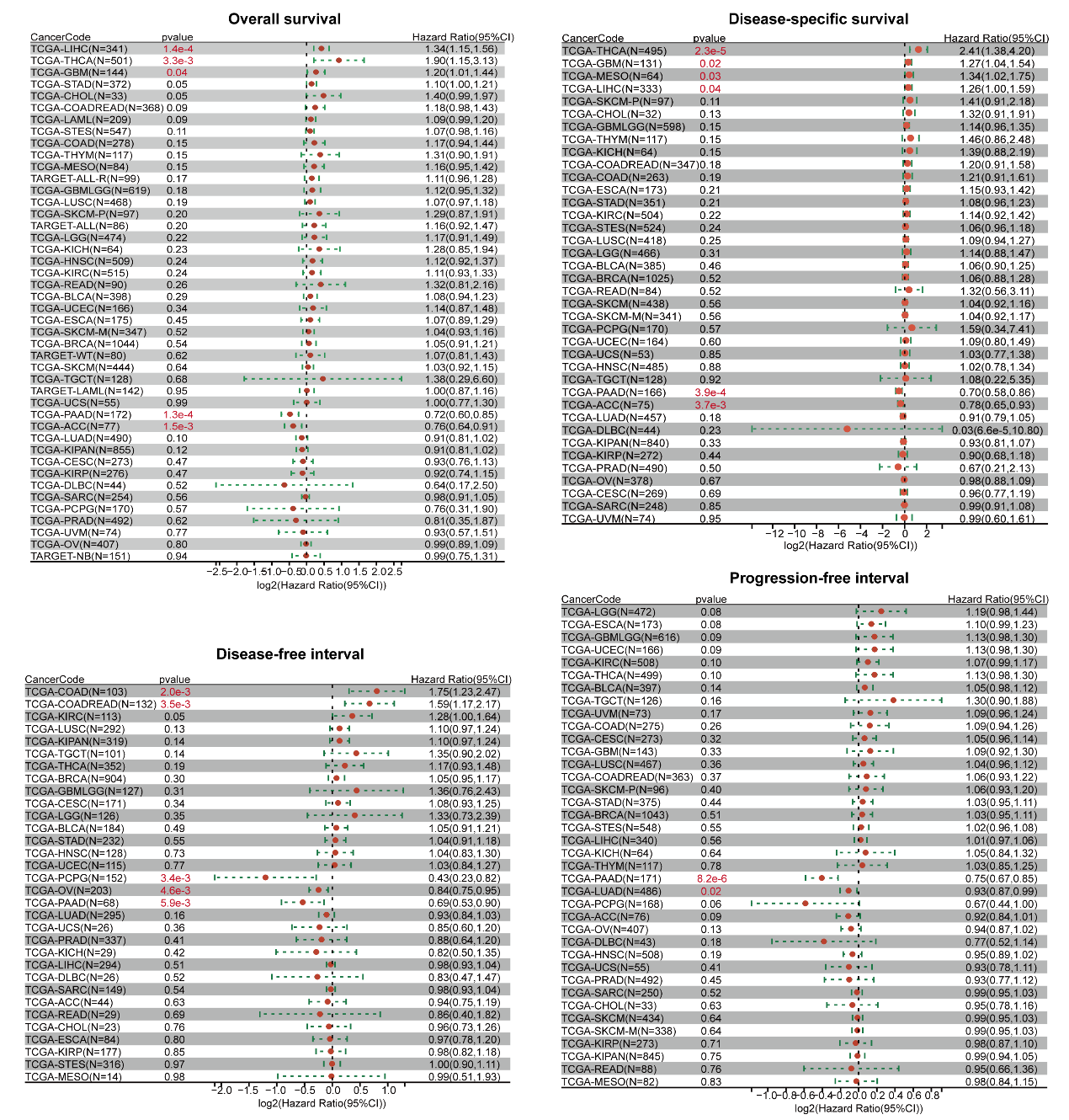


# Fig. S1 Survival analysis of KCNH2 expression in pan-cancer by univariate Cox regression model. HR > 1 indicates that it serves as a risk factor for patients' survival. 95%CI，95% confidence interval.

**
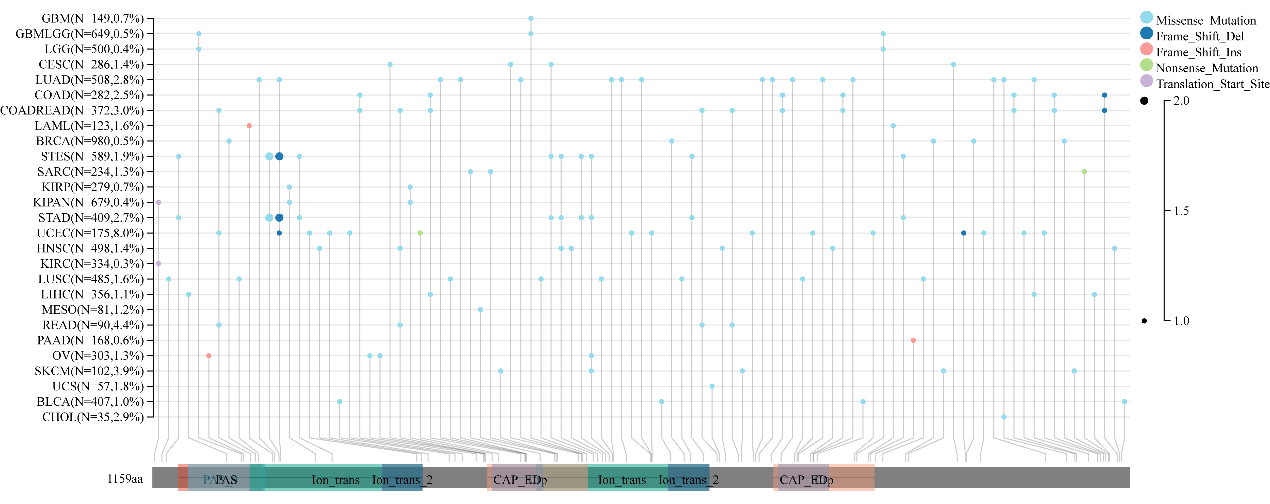
**

# Fig. S2 Mutational landscape of KCNH2 full-length channel in different tumor types. The hERG full-length channel protein contains 1159 amino acids, with the majority of the single nucleotide variant (SNV) sites localized to its transmembrane fragments.

**
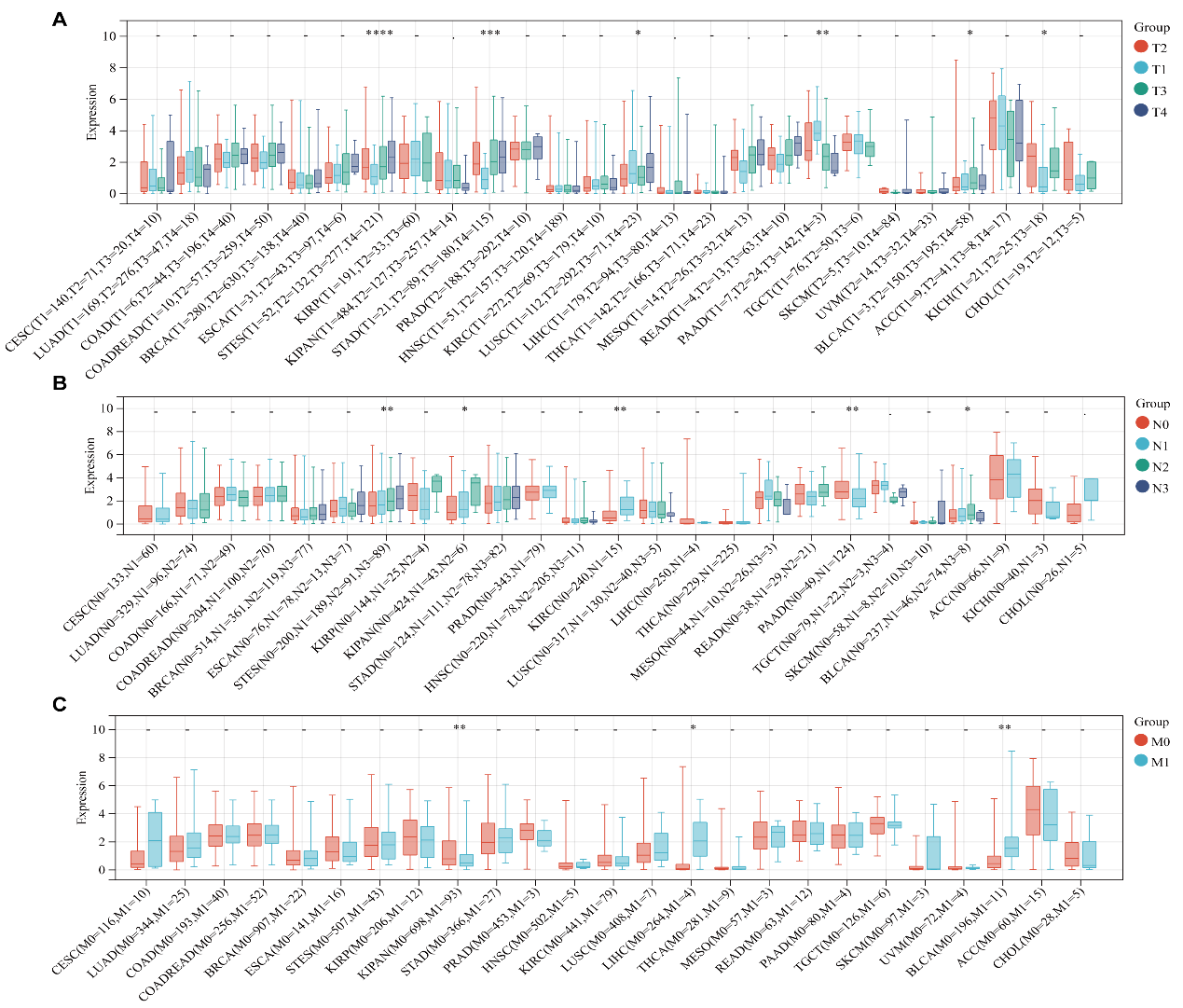
**

# Fig. S3 KCNH2 expression levels in different tumor TNM stages. (A) T staging*.* (B) N staging. (C) M staging. * *P*<0.05, ** *P*<0.01, *** *P*<0.001, **** *P*<0.0001, ^-^ no significance.


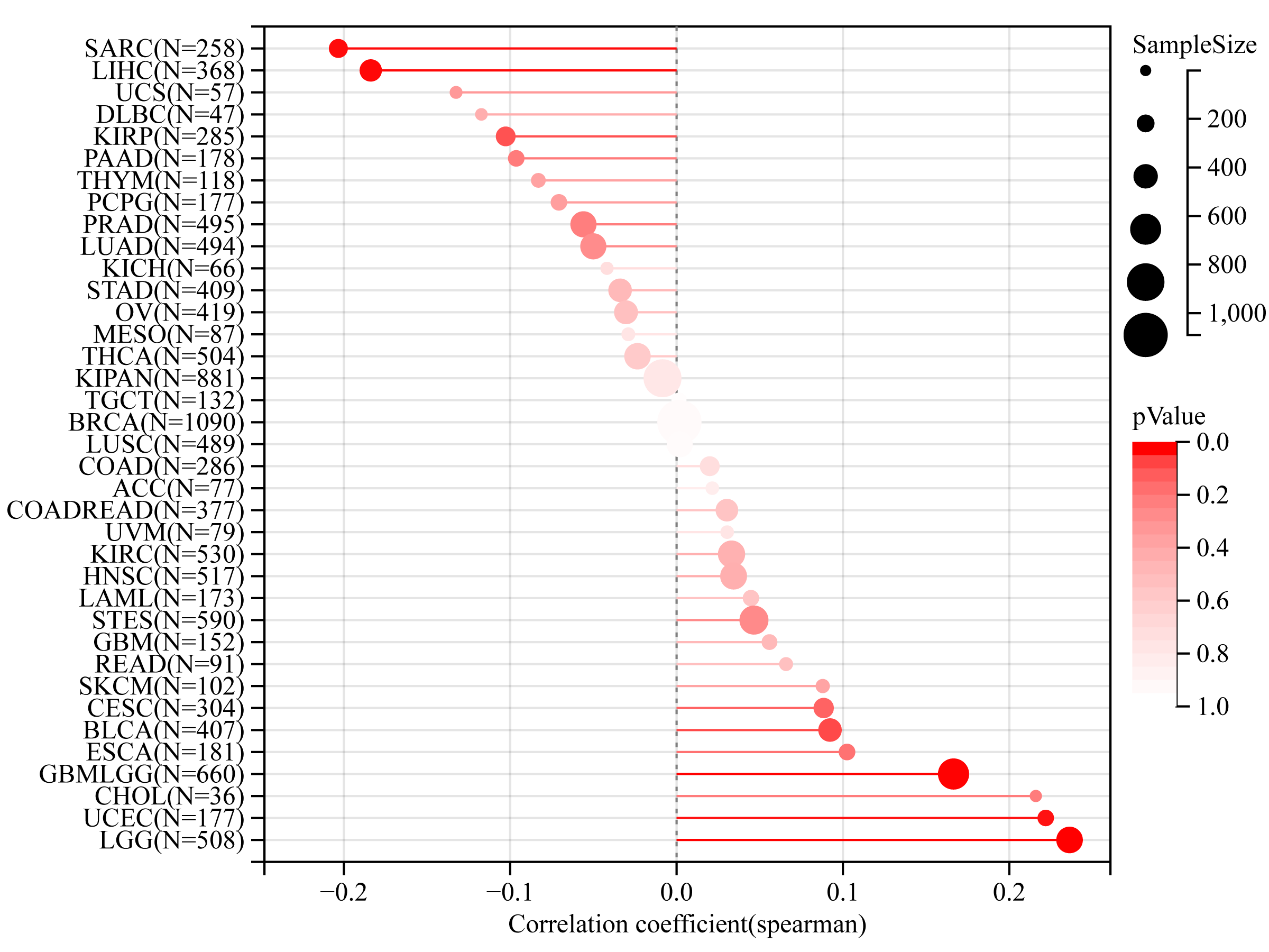


# Fig. S4 Correlation between age and KCNH2 expression levels in different cancers.

# Tab. S1 Correlation of KCNH2 expression with tumor genomic heterogeneity.

| **Analysis** | **Positive correlation** | | | **Negative correlation** | | |
| --- | --- | --- | --- | --- | --- | --- |
|  | Cancer | R | P value | Cancer | R | P |
| **TMB** | LAML | 0.255 | 4.28E-03 | COADREAD | -0.116 | 2.50E-02 |
|  | LGG | 0.269 | 8.54E-10 | STES | -0.159 | 9.72E-05 |
|  | GBMLGG | 0.184 | 2.19E-06 | SARC | -0.182 | 5.15E-03 |
|  |  |  |  | STAD | -0.25 | 2.74E-07 |
|  |  |  |  | UCEC | -0.193 | 1.04E-02 |
|  |  |  |  | PRAD | -0.297 | 1.60E-11 |
|  |  |  |  | THCA | -0.193 | 1.04E-02 |
|  |  |  |  | PAAD | -0.16 | 3.74E-02 |
|  |  |  |  | ACC | -0.366 | 1.05E-03 |
|  |  |  |  | DLBC | -0.475 | 2.91E-03 |
| **MATH** | LUAD | 0.146 | 9.18E-04 | SARC | -0.129 | 4.74E-02 |
|  | COAD | 0.228 | 1.07E-04 | THCA | -0.108 | 1.69E-02 |
|  | COADREAD | 0.215 | 2.69E-05 |  |  |  |
|  | BRCA | 0.077 | 1.58E-02 |  |  |  |
|  | STAD | 0.099 | 4.53E-02 |  |  |  |
|  | UCEC | 0.281 | 1.64E-04 |  |  |  |
|  | HNSC | 0.131 | 3.26E-03 |  |  |  |
|  | LIHC | 0.309 | 2.47E-09 |  |  |  |
|  | SKCM | 0.223 | 2.41E-02 |  |  |  |
|  | BLCA | 0.115 | 1.94E-02 |  |  |  |
|  | ACC | 0.268 | 1.82E-02 |  |  |  |
| **MSI** | KICH | 0.279 | 2.31E-02 | STES | -0.123 | 2.53E-03 |
|  | ACC | 0.368 | 9.79E-04 | SARC | -0.166 | 8.14E-03 |
|  | KIPAN | 0.178 | 2.57E-06 | STAD | -0.158 | 1.21E-03 |
|  | GBM | 0.181 | 2.59E-02 | HNSC | -0.105 | 1.80E-02 |
| **NEO** | KIRC | 0.114 | 4.17E-02 | COAD | -0.203 | 1.90E-03 |
|  |  |  |  | COADREAD | -0.179 | 1.78E-03 |
|  |  |  |  | UCEC | -0.204 | 1.22E-02 |

# Tab. S2 Relationship between KCNH2 expression and immune infiltration by stromal, immune, and ESTIMATE scores.

| **Cancer types** | | **StromalScore** | | **ImmuneScore** | | **ESTIMATEScore** | |
| --- | --- | --- | --- | --- | --- | --- | --- |
|  | R | | P value | R | P value | R | P value |
| TCGA-KIPAN(N=878) | -0.358 | | 5.24E-28 | -0.322 | 1.22E-22 | -0.361 | 2.14E-28 |
| TCGA-THCA(N=503) | 0.367 | | 1.83E-17 | 0.303 | 3.63E-12 | 0.355 | 2.16E-16 |
| TCGA-SARC(N=258) | -0.421 | | 1.68E-12 | -0.395 | 4.41E-11 | -0.441 | 1.11E-13 |
| TCGA-LGG(N=504) | -0.301 | | 5.30E-12 | -0.273 | 4.68E-10 | -0.291 | 2.53E-11 |
| TCGA-GBMLGG(N=656) | -0.247 | | 1.51E-10 | -0.238 | 6.73E-10 | -0.246 | 1.74E-10 |
| TCGA-PRAD(N=495) | 0.444 | | 2.38E-25 | 0.231 | 2.08E-07 | 0.346 | 2.09E-15 |
| TARGET-WT(N=80) | -0.330 | | 2.78E-03 | -0.486 | 4.96E-06 | -0.449 | 2.96E-05 |
| TCGA-CESC(N=291) | 0.044 | | 4.56E-01 | -0.239 | 3.80E-05 | -0.129 | 2.72E-02 |
| TCGA-GBM(N=152) | -0.304 | | 1.38E-04 | -0.311 | 9.63E-05 | -0.308 | 1.14E-04 |
| TCGA-LUAD(N=500) | -0.072 | | 1.07E-01 | -0.141 | 1.53E-03 | -0.119 | 7.78E-03 |
| TCGA-SKCM(N=452) | 0.207 | | 8.89E-06 | 0.135 | 4.02E-03 | 0.177 | 1.57E-04 |
| TCGA-LIHC(N=363) | 0.159 | | 2.34E-03 | 0.149 | 4.55E-03 | 0.167 | 1.42E-03 |
| TCGA-UVM(N=79) | 0.408 | | 1.90E-04 | 0.313 | 4.92E-03 | 0.358 | 1.21E-03 |
| TCGA-KIRP(N=285) | -0.212 | | 3.21E-04 | -0.166 | 4.93E-03 | -0.192 | 1.13E-03 |
| TCGA-SKCM-M(N=351) | 0.198 | | 1.84E-04 | 0.117 | 2.90E-02 | 0.161 | 2.48E-03 |
| TCGA-TGCT(N=132) | -0.005 | | 9.51E-01 | -0.188 | 3.07E-02 | -0.146 | 9.42E-02 |
| TCGA-OV(N=417) | -0.088 | | 7.20E-02 | -0.095 | 5.31E-02 | -0.100 | 4.03E-02 |
| TARGET-ALL-R(N=99) | 0.249 | | 1.29E-02 | 0.193 | 5.60E-02 | 0.213 | 3.42E-02 |
| TCGA-MESO(N=85) | -0.105 | | 3.37E-01 | -0.203 | 6.29E-02 | -0.198 | 6.96E-02 |
| TCGA-KICH(N=65) | -0.209 | | 9.53E-02 | -0.225 | 7.21E-02 | -0.236 | 5.87E-02 |
| TCGA-HNSC(N=517) | 0.212 | | 1.12E-06 | 0.077 | 8.10E-02 | 0.155 | 4.12E-04 |
| TCGA-UCEC(N=178) | -0.068 | | 3.68E-01 | -0.131 | 8.18E-02 | -0.106 | 1.60E-01 |
| TARGET-NB(N=153) | -0.365 | | 3.41E-06 | -0.139 | 8.55E-02 | -0.275 | 5.68E-04 |
| TARGET-LAML(N=142) | -0.229 | | 6.10E-03 | -0.140 | 9.65E-02 | -0.184 | 2.86E-02 |
| TCGA-BRCA(N=1077) | -0.178 | | 4.26E-09 | -0.041 | 1.82E-01 | -0.112 | 2.24E-04 |
| TCGA-STES(N=569) | 0.294 | | 8.03E-13 | 0.056 | 1.84E-01 | 0.186 | 7.61E-06 |
| TCGA-DLBC(N=46) | 0.061 | | 6.88E-01 | 0.198 | 1.87E-01 | 0.121 | 4.22E-01 |
| TCGA-UCS(N=56) | 0.260 | | 5.29E-02 | -0.169 | 2.14E-01 | 0.043 | 7.56E-01 |
| TCGA-STAD(N=388) | 0.279 | | 2.30E-08 | -0.057 | 2.59E-01 | 0.118 | 2.03E-02 |
| TCGA-BLCA(N=405) | 0.310 | | 1.81E-10 | 0.048 | 3.36E-01 | 0.188 | 1.45E-04 |
| TCGA-CHOL(N=36) | -0.119 | | 4.91E-01 | -0.155 | 3.65E-01 | -0.137 | 4.24E-01 |
| TCGA-READ(N=91) | 0.262 | | 1.22E-02 | -0.077 | 4.71E-01 | 0.097 | 3.62E-01 |
| TCGA-PAAD(N=177) | -0.026 | | 7.29E-01 | -0.051 | 5.02E-01 | -0.047 | 5.37E-01 |
| TCGA-ESCA(N=181) | 0.183 | | 1.37E-02 | 0.050 | 5.07E-01 | 0.127 | 8.94E-02 |
| TCGA-ACC(N=77) | -0.036 | | 7.57E-01 | 0.073 | 5.29E-01 | 0.037 | 7.52E-01 |
| TCGA-LAML(N=214) | 0.246 | | 2.79E-04 | 0.040 | 5.63E-01 | 0.139 | 4.19E-02 |
| TCGA-SKCM-P(N=101) | 0.034 | | 7.38E-01 | -0.056 | 5.78E-01 | -0.027 | 7.86E-01 |
| TCGA-THYM(N=118) | -0.250 | | 6.29E-03 | 0.039 | 6.73E-01 | -0.155 | 9.32E-02 |
| TCGA-KIRC(N=528) | 0.130 | | 2.68E-03 | -0.017 | 7.00E-01 | 0.049 | 2.63E-01 |
| TARGET-ALL(N=86) | 0.056 | | 6.06E-01 | 0.041 | 7.05E-01 | 0.070 | 5.21E-01 |
| TCGA-COADREAD(N=373) | 0.196 | | 1.39E-04 | -0.014 | 7.84E-01 | 0.099 | 5.56E-02 |
| TCGA-PCPG(N=177) | -0.055 | | 4.68E-01 | -0.017 | 8.26E-01 | -0.030 | 6.89E-01 |
| TCGA-LUSC(N=491) | 0.059 | | 1.93E-01 | 0.002 | 9.58E-01 | 0.026 | 5.69E-01 |
| TCGA-COAD(N=282) | 0.169 | | 4.51E-03 | 0.001 | 9.82E-01 | 0.094 | 1.16E-01 |
